# Macular Curvature in Children Born Preterm With and Without ROP – Results From the Gutenberg Prematurity Study Young

**DOI:** 10.1167/iovs.67.10.27

**Published:** 2026-08-11

**Authors:** Achim Fieß, Sandra Gißler, Alica Hartmann, Dana Gattermayer, Maria P. De la Ossa Maury, Christina Korb, Eva Mildenberger, Bernhard Stoffelns, Panagiotis Laspas, Stephanie Grabitz, Alexander K. Schuster

**Affiliations:** 1 Department of Ophthalmology, University Medical Center of the Johannes Gutenberg University Mainz, Mainz, Germany; 2 Division of Neonatology, Department of Pediatrics, University Medical Center of the Johannes Gutenberg University Mainz, Mainz, Germany

**Keywords:** gestational age (GA), retinopathy of prematurity (ROP), macular curvature, dome-shaped macula (DSM), epidemiology

## Abstract

**Purpose:**

This study investigated the associations of prematurity, retinopathy of prematurity (ROP), and ocular geometry with the macular curvature coefficient in children and adolescents.

**Methods:**

The Gutenberg Prematurity Study Young is a retrospective cohort study of preterm and full-term participants aged 4 to 17 years with a prospective ophthalmologic examination. The macular curvature coefficient in the foveal scan imaged with optical coherence tomography (OCT) and its associations with gestational age (GA), birth weight (BW) percentile, ROP occurrence and treatment, and other perinatal factors were investigated. These were evaluated in linear regression analyses. A second analysis assessed the association with ocular geometry.

**Results:**

In the present study, 453 eyes of 234 term-born participants and 906 eyes of 469 preterm-born participants were examined (mean age = 11.8 years, 375 girls). In multivariable analyses for perinatal parameters, ROP treatment (B = −57.82, *P* < 0.001) and perinatal adverse events (B = −15.3, *P* = 0.01) showed a negative association with the macular curvature coefficient. Regarding ocular geometry, spherical equivalent was negatively associated with the macular curvature coefficient after adjustment for age and sex (B = −6.19, *P* = 0.004). In a sensitivity analysis, subfoveal choroidal thickness was negatively associated with the macular curvature coefficient (B = −0.10, *P* = 0.001).

**Conclusions:**

Our study showed that ROP treatment and perinatal adverse events were independently linked to a flatter macular shape in childhood and adolescence. The age-related growth of axial length leads to a larger macular curvature coefficient. Subfoveal choroidal thickness is involved in macular shape development at this age.

Preterm birth contributes to morphological ocular alterations that may persist from infancy into adulthood. Changes in axial length, retinal thickness, and foveal development have been detected in both children and adults,^1^^–^^4^ potentially increasing susceptibility to age-related retinal diseases such as age-related macular degeneration (AMD) in later life.^5^

Most studies addressing macular curvature refer to “dome-shaped macula/maculopathy” (DSM), first described by Gaucher et al. as a protrusion of the macula toward the vitreous cavity, typically associated with posterior staphyloma and myopia.^6^ Legocki et al. reported a higher prevalence of DSM in preterm infants with ROP, particularly with pre-plus/plus-disease.^7^ In adults aged 18 to 52 years with ROP treated with cryo- and laser coagulation, a flatter shaped macular curvature was observed.^8^ Further data for children and adolescents remain limited.

Alterations in macular curvature without specification of DSM have been described in relation to retinal diseases. In individuals with retinitis pigmentosa (RP), a significantly lower macular curvature index has been reported, indicating a deeper concave shape^9^^,^^10^ and linked to the *RPGR* gene (Retinitis-pigmentosa-GTPase-Regulator).^11^ The curvature index was also positively correlated with age and negatively with axial length in patients with RP.^9^ Macular curvature has further been associated with ocular geometry, including axial length, posterior segment length, subfoveal choroidal thickness, age, choroidal and scleral thickness,^8^^,^^12^ foveal curvature,^13^ and posterior staphyloma, especially in highly myopic eyes.^14^^,^^15^ In a UK analysis, macular curvature was related to demographic, ocular, and infancy factors, with dome-shaped macula more frequent in individuals with high refractive error, including myopia and hypermetropia.^16^

Most studies have focused on adults. Limited pediatric data indicate associations among macular curvature, anisomyopia, and retinal microvascular parameters^17^ but not necessarily between DSM and high myopia.^18^

Therefore, this study investigates the macular curvature coefficient in children aged 4 to 17 years and its association with factors related to preterm birth, and which ocular geometric factors may be involved in its changes.

## Materials and Methods

### Study Population

The Gutenberg Prematurity Eye Study Young (GPESY) is based at the University Medical Center of the Johannes Gutenberg University Mainz (UMCM) in Germany and examined a single-center cohort of 4 to 17-year-old children and adolescents born preterm or at term (between 2003 and 2018) for the effects of prematurity and related factors on the eyes.^19^^,^^20^ The study consisted of a combined retrospective and prospective approach. Perinatal history was assessed retrospectively by reviewing medical records stored in the UMCM, whereas the prospective part consisted of an extensive ophthalmologic examination. Potential study participants were selected by an algorithm inviting every former preterm newborn with a gestational age (GA) at birth ≤32 weeks and every third randomly chosen preterm newborn with a GA of 33 to 36 weeks. Additionally, study controls of 8 randomly selected full-term subjects (4 male subjects and 4 female subjects for each month of the calendar) with a birth weight (BW) between the 10th and 90th percentile were also recruited. A flow chart for eligibility and group sizes as well as sizes of subgroups is displayed in Supplementary Figure S1. The participants were examined between 2021 and 2023 and surveyed to gain insight into their medical history. Written informed consent was obtained from all participants and their legal guardians before their study entry. The GPESY complies with Good Clinical Practice (GCP), Good Epidemiological Practice (GEP), and the ethical principles of the Declaration of Helsinki. The study protocol and study documents were approved by the local ethics committee of the Medical Chamber of Rhineland-Palatinate, Germany (reference no. 2021–15830; original vote: May 5, 2021, latest update: January 19, 2022).

### Assessment of Pre-, Peri-, and Postnatal Medical History

Medical records at the UMCM were reviewed for the following data: GA (weeks), BW (kg), presence of ROP, stage of ROP, ROP treatment, placental insufficiency, pre-eclampsia, breastfeeding, maternal smoking during pregnancy, and perinatal adverse events. Perinatal adverse events were defined according to the German query for quality control of the neonatal clinics: occurrence of intraventricular hemorrhage (at least grade 3 or parenchymal hemorrhage), occurrence of necrotizing enterocolitis, and moderate or severe bronchopulmonary dysplasia were summarized as adverse events.^21^ BW percentiles were calculated according to Voigt et al.^22^

### Categorization

Participants were categorized in 6 groups, consisting of term-born children with a GA at birth ≥37 completed weeks (group 1), participants born preterm with GA of 33 to 36 weeks without ROP (group 2), GA of 29 to 32 weeks without ROP (group 3), GA ≤28 weeks without ROP (group 4), GA ≤32 weeks and non-treated ROP (group 5), and treated ROP (group 6). In the case that only one eye was affected with ROP, the other non-ROP eye was excluded from the analysis.

### Ophthalmologic Examination

The detailed ophthalmologic examination included testing of distant corrected visual acuity (DCVA) with ARK 1s (NIDEK; Oculus, Wetzlar, Germany) and the measurement of central corneal thickness, corneal radius, anterior chamber depth, posterior segment length, and axial length with LenStar (Lenstar LS900; Haag-Streit, Bern, Switzerland). Additionally, intraocular pressure was measured with a non-contact tonometer (NT 2000; Nidek Co., Japan). According to medical literature, visual acuity was converted from decimal to logMAR.^23^

### Optical Coherence Tomography

For macular optical coherence tomography (OCT) imaging, the spectral-domain OCT (SD-OCT; OCT2; Heidelberg Engineering, Heidelberg, Germany) was used for a block scan 15 × 15 degrees in the enhanced depth imaging (EDI)-mode and 7.7 mm corneal curvature. The automatic segmentation was performed by a research software tool of the Heidelberg Eye Explorer Software (HEYEX; SPX, Heidelberg Engineering, Heidelberg, Germany) which segmented the retinal thickness of the macula as well as each retinal layer separately. This included measurements in microns in the fovea of the total retinal (TR) layer. The foveal center was defined as the deepest foveal depression and the foveal retinal thickness was defined as the minimum foveal retinal thickness in the foveal center. All SD-OCT measurements were performed in a darkened room on mydriatic eyes. Each scan was checked for decentration or layer segmentation errors, and eyes with such errors were excluded. Thus, the present study only includes high quality images with ideal centration as well as high signal strength of >15 decibels (dB).

### Definition of the Macular Curvature

After Bruch's membrane (BM) segmentation (by HEYEX-Software, Heidelberg Engineering, Heidelberg, Germany) and verification of scan centration, central foveal OCT scans with 1:1 µm scaling were exported for macular curvature analysis. In cases of good scan quality but inadequate BM segmentation, the BM segmentation was manually corrected. The images were binarized using ImageJ software (National Institutes of Health, Bethesda, MD, USA), whereby all red pixels were converted to black and all remaining pixels to white. The coordinates of the black pixels were subsequently exported from the software. Macular curvature was then calculated from the black pixel coordinates corresponding to the BM segmentation line using a quadratic polynomial fit (x^2^) and the second derivative thereof was used as curvature coefficient (see the Fig.). A quadratic model was chosen because the OCT scans did not include wide-field imaging, placing potential inflection points for dome height measurements outside the scan range. Negative coefficients indicated protrusion toward the vitreous cavity as expected in DSM. Curvature coefficients were multiplied by 10^6^ to facilitate interpretation and *R*^2^ values were assessed to ensure fit quality.

**Figure.**
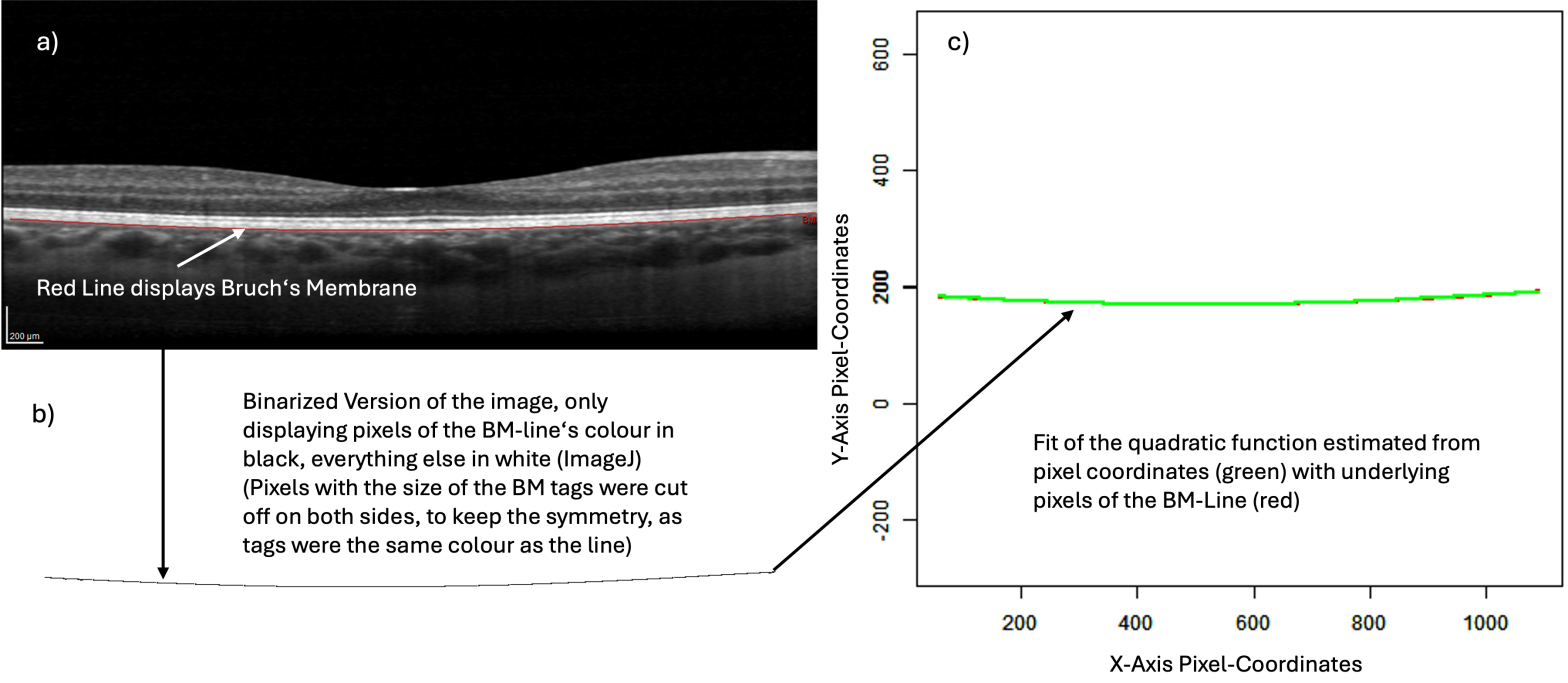
**Exporting curvature data from OCT images.** (**a**) OCT image displaying the Bruch's Membrane as automatically traced by the Software (*red line*). (**b**) Binarized version of the OCT image, only displaying pixels of the BM-line's color in *black*, everything else in *white* with ImageJ software. Sixty pixels were taken off on each side of the image to prevent accidental inclusion of the red “BM”-tag in the derived pixel coordinates. (**c**) The quadratic fit is displayed in *green* whereas the original pixel coordinates are only slightly shown underneath in a *red* color.

### Inclusion and Exclusion Criteria

In the present analysis, only participants with a sufficient quality of the central foveal SD-OCT scan and a sufficient polynomial fit (mean *R*^2^ = 0.99 of all OCT scans, minimal *R*^2^ = 0.75) of BM lines were included in the analysis.

### Covariables

Two different aspects of macular curvature were evaluated: (1) factors that may affect the macular curvature with a specific focus on perinatal parameters including GA (weeks), BW (kg), BW percentile, ROP, ROP treatment, placental insufficiency, pre-eclampsia, maternal smoking, breastfeeding, and perinatal adverse events; and (2) the relationship of macular curvature with geometric features of the eye, such as central corneal thickness (µm), anterior chamber depth (µm), lens thickness (µm), posterior segment length (µm), foveal retinal thickness, and subfoveal choroidal thickness.

### Statistical Analysis

The main outcome measure consisted of the macular curvature. Macular curvature was defined as the second derivative of a quadratic fit, gained from exporting X- and Y-coordinates from BM-pixels in 1:1 µm-scaled OCT scans where the BM-line had been segmented automatically by HEYEX research-software (HEYEX; SPX, Heidelberg Engineering, Heidelberg, Germany). The quadratic function was fitted using R software (R Core Team, 2023). Descriptive statistics were stratified by groups one to six. Absolute and relative frequencies were calculated for dichotomous parameters and mean and standard deviation were calculated for approximately normally distributed variables (otherwise median and interquartile range). One-way ANOVA was used to compare continuous and approximately normally distributed variables and χ^2^ test for dichotomous variables. For non-normally distributed variables, the Kruskal-Wallis test was used to compare continuous variables. For association analyses, linear regression with generalized estimating equations (GEEs) was used in a complete-case analysis set to account for inter-eye correlations within each participant.

The analysis was divided into two parts, one analyzing the impact of perinatal factors, the other the association with ocular geometrical factors. OCT-related optical distortions might affect the image display, which is why, apart from correcting for sex and age, we additionally adjusted our perinatal models for corneal radius (mm) and axial length (mm).

The analysis of the impact of perinatal factors on the macular curvature coefficient consisted of univariable analyses adjusted for age (years) and sex (female children) for the following factors: GA (weeks), BW (kilograms), BW percentile, ROP (yes), ROP-treatment (yes), perinatal adverse events (yes), maternal smoking during pregnancy (yes), pre-eclampsia (yes), placental insufficiency (yes), and breastfeeding (yes). In a second model, these factors were additionally adjusted for corneal radius (mm) and axial length (mm). Model 3 was a multivariable model including associated parameters from model 1 and/or model 2, apart from ROP-treatment, due to high collinearity with ROP. In model 4, a multivariable model with all parameters associated with the macular curvature coefficient in model 1 and/or model 2, ROP was excluded due to high collinearity with ROP-treatment. Collinearity in models 3 and 4 was assessed using variance inflation factors (VIFs). All values were below 3, indicating no relevant collinearity.

The analysis of the association with geometric ocular features excluded participants treated for ROP and consisted of univariable analyses for the following factors: anterior chamber depth (mm), lens thickness (mm), posterior segment length (mm), foveal retinal thickness (mm), and spherical equivalent (D; model 1). In model 2, all factors were adjusted for age (years) and sex (female children).

Measurements of subfoveal choroidal thickness had a relevant number of missing information, which is why it was only assessed in a sensitivity analysis, consisting of the same models as the geometric ocular features but including subfoveal choroidal thickness in a complete case analysis.

As this is an explorative study, a significance level was not defined and no adjustment for multiple testing was conducted. Thus, *P* values are reported only for descriptive purposes and should be interpreted with caution.^24^ Calculations were performed using R software (R Core Team, 2023; R: A language and environment for statistical computing; R Foundation for Statistical Computing, Vienna, Austria, URL https://www.R-project.org/, R version 4.3.1, 2023-06-16).

## Results

### Participant Characteristics

In the present study, 453 eyes of 234 term-born participants and 906 eyes of 469 preterm participants were examined (mean age = 11.8 years, 375 girls). The group characteristics are displayed in Table 1. In the group treated for ROP, seven participants were treated with laser coagulation (12 eyes) and one was treated with intravitreal application of anti-vascular endothelial growth factors (2 eyes). No cryocoagulation was performed.

**Table 1.** Characteristics of the GPESY Sample (*n* = 703), Stratified by Study Groups

	Group 1 GA ≥37 Wk	Group 2 GA 33–36 Wk No ROP	Group 3 GA 29–32 Wk No ROP	Group 4 GA ≤28 Wk No ROP	Group 5 GA ≤32 Wk ROP Without Treatment	Group 6 GA ≤32 Wk ROP With Treatment
Participants, *n*/eyes, *n*	234/453	215/419	149/288	61/116	36/69	8/14
Sex, F	127 (54.3)	108 (50.2)	80 (53.7)	37 (60.7)	19 (52.8)	4 (50.0)
Age, y, mean (SD)	11.65 (3.33)	11.33 (3.64)	12.12 (3.64)	11.79 (3.26)	14.39 (3.77)	11.50 (3.42)
Gestational age, wk, mean (SD)	39.00 (1.27)	34.65 (1.02)	30.83 (1.06)	26.16 (1.49)	26.86 (2.57)	24.12 (0.99)
Birthweight, kg, mean (SD)	3.38 (0.39)	2.33 (0.40)	1.56 (0.36)	0.83 (0.23)	0.95 (0.36)	0.62 (0.17)
Birthweight <1500 g, yes (%)	0 (0.0)	6 (2.8)	68 (45.6)	61 (100.0)	34 (94.4)	8 (100.0)
Birthweight <1000 g, yes (%)	0 (0.0)	0 (0.0)	9 (6.0)	47 (77.0)	24 (66.7)	8 (100.0)
Birthweight percentile, mean (SD)	45.49 (24.46)	32.94 (22.11)	40.91 (22.47)	36.34 (25.73)	39.06 (25.67)	13.38 (9.91)
ROP, yes (%)	0 (0.0)	0 (0.0)	0 (0.0)	0 (0.0)	36 (100.0)	8 (100.0)
ROP stage (1/2/3)	(0/0/0)	(0/0/0)	(0/0/0)	(0/0/0)	(18/10/8)	(0/1/7)
Perinatal adverse events, yes* (%)	0 (0.0)	1 (0.5)	7 (4.7)	24 (39.3)	15 (41.7)	7 (87.5)
Intraventricular hemorrhage^†^, yes (%)	0 (0.0)	0 (0.0)	0 (0.0)	6 (9.8)	1 (2.8)	0 (0.0)
Necrotizing enterocolitis, yes (%)	0 (0.0)	1 (0.5)	4 (2.7)	2 (3.3)	1 (2.8)	2 (25.0)
Bronchopulmonary dysplasia^‡^, yes (%)	0 (0.0)	0 (0.0)	3 (2.0)	22 (36.1)	16 (44.4)	7 (87.5)
Pre-eclampsia, yes (%)	4 (1.7)	20 (9.3)	28 (18.8)	11 (18.0)	8 (22.2)	1 (12.5)
Placental insufficiency, yes (%)	0 (0.0)	8 (3.7)	6 (4.0)	6 (9.8)	2 (5.6)	0 (0.0)
HELLP Syndrome, yes (%)	0 (0.0)	6 (2.8)	17 (11.4)	5 (8.2)	1 (2.8)	0 (0.0)
Gestational diabetes (%)	28 (12.0)	17 (7.9)	18 (12.1)	3 (4.9)	1 (2.8)	0 (0.0)
Maternal smoking during pregnancy^§^, yes (%)	11 (4.7)	7 (3.3)	5 (3.4)	4 (6.6)	5 (13.9)	2 (25.0)
Breastfeeding, yes (%)	203 (86.8)	162 (75.3)	108 (72.5)	42 (68.9)	16 (44.4)	1 (12.5)
Ocular parameters						
Visual acuity, LogMAR, OD (median [IQR])	0.00 [0.00, 0.10]	0.00 [0.00, 0.10]	0.00 [0.00, 0.10]	0.00 [0.00, 0.10]	0.00 [0.00, 0.00]	0.20 [0.15, 0.20]
Visual acuity, LogMAR OS (median [IQR])	0.00 [0.00, 0.10]	0.10 [0.00, 0.20]	0.00 [0.00, 0.10]	0.00 [0.00, 0.10]	0.00 [0.00, 0.10]	0.10 [0.10, 0.40]
Spherical equivalent, D, OD, mean (SD)	0.27 (1.38)	0.36 (1.39)	0.36 (1.51)	0.24 (1.56)	0.87 (1.36)	−1.10 (2.89)
Spherical equivalent, D, OS, mean (SD)	0.30 (1.40)	0.39 (1.43)	0.49 (1.58)	0.46 (1.85)	0.87 (1.44)	−1.18 (2.98)
Intraocular pressure, mm Hg, OD, mean (SD)	17.09 (3.11)	16.33 (2.64)	16.35 (3.00)	18.34 (4.07)	17.65 (4.36)	16.40 (2.97)
Intraocular pressure, mm Hg, OS, mean (SD)	16.52 (2.94)	15.76 (2.73)	15.67 (3.03)	17.03 (3.38)	17.07 (4.36)	15.55 (3.95)

D, diopter; GA, gestational age; HELLP, hemolysis, elevated liver enzymes, low platelet; IQR, interquartile range; kg, kilograms; *n*, number; OD, right eye; OS, left eye; ROP, retinopathy of prematurity; SD, standard deviation; wk, weeks; y, years.

*Perinatal adverse events were defined as the occurrence of intraventricular hemorrhage (^†^at least grade 3 or parenchymal hemorrhage) and/or occurrence of necrotizing enterocolitis and/or bronchopulmonary dysplasia (^‡^moderate or severe).

§ Maternal smoking during pregnancy.

**Table 2.** Geometrical Parameters of the Eye and Curvature Parameters for the GPESY Sample (*n* = 703) for Each Study Group

	Group 1 GA ≥37 Wk	Group 2 GA 33–36 Wk No ROP	Group 3 GA 29–32 Wk No ROP	Group 4 GA ≤28 Wk No ROP	Group 5 GA ≤32 Wk ROP Without Treatment	Group 6 GA ≤32 Wk ROP With Treatment
Eyes, *n*, OD/OS	228/225	209/210	144/144	60/56	34/35	7/7
Curvature coefficient, mean (SD) OD	78.52 (39.51)	82.41 (39.00)	82.37 (38.04)	72.73 (44.65)	74.07 (40.63)	−10.07 (54.35)
Curvature coefficient, mean (SD), OS	78.70 (39.26)	79.55 (39.83)	80.88 (41.65)	66.02 (41.64)	68.36 (47.17)	14.66 (45.82)
Negative curvature, yes (%), OD	5 (2.19)	2 (0.96)	2 (1.39)	4 (6.67)	0 (0.0)	4 (57.14)
Negative curvature, yes (%), OS	2 (0.89)	4 (1.90)	3 (2.08)	3 (5.36)	2 (5.71)	2 (28.57)

D, diopter; GA, gestational age; *n*, number; OD, right eye; OS, left eye; ROP, retinopathy of prematurity; SD, standard deviation; wk, week; y, years.

### Descriptive Curvature Measures

The descriptive curvature measures are displayed in Table 2. The macular curvature coefficient was descriptively smallest in the group treated for ROP, both in the right and left eyes. The right eyes even showed a negative mean curvature coefficient, indicating a protrusion toward the vitreous cave. Therefore, the prevalence of negative curvature coefficient values was assessed between the groups and significant differences (*P* < 0.001) were found, with eyes treated for ROP having the highest prevalences in negative curvature coefficients (ROP-treated eyes: OD = 57% and OS = 29%), respectively.

### Association Analyses of Perinatal History

The association analyses for perinatal history and the curvature coefficients are displayed in Table 3^4^. GA, BW, ROP, treatment of ROP, and a history of perinatal adverse events were associated with the macular curvature coefficient in univariable analyses and after adjustment of age and sex, as well as after additional adjustment of axial length and corneal radius. However, when assessed in a multivariable model, only ROP-treatment and perinatal adverse events showed an association with the curvature coefficient in a negative direction, indicating that children with treated ROP or who have experienced perinatal adverse events display a smaller macular curvature coefficient. Perinatal adverse events consisted mainly of participants with bronchopulmonary dysplasia, however, due to low numbers in other categories of perinatal adverse events, no further analysis was conducted.

**Table 3.** Association Analyses of Macular Curvature With Perinatal Factors

Curvature	Model 1 (95% CI)	*P* Value	Model 2 (95% CI)	*P* Value	Model 3 (95% CI)	*P* Value	Model 4 (95% CI)	*P* Value
Gestational age, wk	0.82 (0.1 to 1.53)	0.03	0.87 (0.13 to 1.61)	0.02	0.02 (−0.85 to 0.88)	0.97	0.17 (−0.64 to 0.98)	0.68
Birthweight, kg	3.75 (0.32 to 7.18)	0.03	4.14 (0.55 to 7.73)	0.02	—	—	—	—
Birthweight percentile	0.08 (−0.05 to 0.22)	0.21	0.09 (−0.05 to 0.23)	0.2	—	—	—	—
ROP, yes	−27.47 (−42.56 to −12.37)	<0.001	−24.59 (−39.14 to −10.0)	<0.001	−17.14 (−33.45 to −0.83)	0.04	—	—
ROP treatment, yes	−74.26 (−103.34 to −45.18)	<0.001	−70.60 (−98.53 to −42.7)	<0.001	—	—	−57.82 (−85.41 to −30.24)	<0.001
Perinatal adverse events, yes*	−25.42 (−37.23 to −13.61)	<0.001	−24.72 (−36.67 to −12.8)	<0.001	−18.35 (−31.85 to −4.85)	0.008	−15.30 (−27.12 to −3.48)	0.01
Pre-eclampsia, yes (%)	−8.82 (−19.00 to 1.36)	0.09	−8.25 (−18.34 to 1.84)	0.11	—	—	—	—
Placental insufficiency, yes (%)	−11.46 (−24.78 to 1.86)	0.09	−11.27 (−24.95 to 2.54)	0.11	—	—	—	—
Maternal smoking during pregnancy,^†^ yes (%)	−11.80 (−27.46 to 3.87)	0.14	−10.23 (−26.23 to 5.77)	0.21	—	—	—	—
Breastfeeding, yes (%)	3.74 (−3.77 to 11.24)	0.33	4.20 (−3.26 to 11.65)	0.27	—	—	—	—

Model 1: Univariable analysis, adjusted for sex and age.

Model 2: Model 1 + additional adjustment for corneal radius and axial length.

Model 3: Multivariable model with all univariably associated parameters and adjustment for age, sex, axial length, and corneal radius, excluding ROP-treatment due to high collinearity with ROP.

Model 4: Multivariable model with all univariably associated parameters and adjustment for age, sex, axial length, and corneal radius, excluding ROP due to high collinearity with ROP-treatment.

Complete case analysis in 1143 eyes from 594 participants.

*Perinatal adverse events were defined as the occurrence of intraventricular hemorrhage (^†^at least grade 3 or parenchymal hemorrhage) and/or occurrence of necrotizing enterocolitis and/or bronchopulmonary dysplasia (moderate or severe).

**Table 4.** Association Analyses of Macular Curvature With Ocular Geometry

Curvature	Model 1 (95% CI)	*P* Value	Model 2 (95% CI)	*P* Value
Sex, F	3.75 (−2.81 to 10.3)	0.26	—	—
Age, y	2.42 (1.41 to 3.43)	<0.001	—	—
Central corneal thickness, µm	−0.09 (−0.19 to 0.01)	0.09	−0.08 (−0.18 to 0.02)	0.10
Corneal radius, mm	−8.96 (−20.1 to 2.2)	0.12	−9.35 (−20.89 to 2.20)	0.11
Anterior chamber depth, mm	−9.24 (−23.6 to 5.1)	0.21	−12.9 (−27.53 to 1.72)	0.08
Lens thickness, µm	1.96 (−17.5 to 21.4)	0.84	5.52 (−13.98 to 25.02)	0.58
Posterior segment length, mm	5.95 (1.33 to 10.6)	0.01	4.58 (−0.69 to 9.86)	0.09
Foveal retinal thickness, µm	0.07 (−0.08 to 0.22)	0.37	0.04 (−0.12 to 0.19)	0.65
Spherical equivalent, D	−7.08 (−11.4 to −2.81)	0.001	−6.19 (−10.42 to −1.95)	0.004

Model 1: Univariable model without adjustment for sex and age.

Model 2: Univariable model with additional adjustment for sex and age.

Complete case analysis of 895 eyes in 525 participants.

### Association Analyses of Ocular Geometry

This study additionally assessed the association of ocular geometry with the macular curvature coefficient (Table 4). In univariable analyses, only posterior segment length was associated with macular curvature. When adjusted for sex and age, the association was not observed. A rather strong association was found between the macular curvature coefficient and spherical equivalent in univariable analysis (B = −7.08, 95% confidence interval [CI] = −11.4 to −2.81, *P* < 0.001) and also after adjustment for age and sex (B = −6.19, 95% CI = −10.42 to −1.95, *P* = 0.004) (Table 4). A descriptive scatterplot additionally depicts this association (see Supplementary Fig. S2).

### Sensitivity Analysis With Inclusion of Subfoveal Choroidal Thickness

As the number of OCT scans with a valid capture and segmentation of the subfoveal choroid was rather small, a separate sensitivity analysis was performed to assess whether the subfoveal choroidal thickness was associated with the macular curvature coefficient (*n* = 382 participants). In both univariable analysis and after adjustment for age and sex, subfoveal choroidal thickness was associated with the macular curvature coefficient (univariable: B = −0.09, 95% CI = −0.16 to −0.03, *P* = 0.003, and adjusted: B = −0.10, 95% CI = −0.17 to −0.04, *P* = 0.001).

## Discussion

This study assessed the association between the macular curvature coefficient and potential perinatal risk factors, as well as ocular geometric parameters. Macular curvature coefficients were derived from the quadratic fit of the OCT B-scan curvature. Descriptive curvature values were lowest in the group with treated ROP. In a multivariable model regarding potential perinatal risk factors, only ROP-treatment and perinatal adverse events remained associated with the curvature coefficient, making it smaller. Regarding associations of ocular geometry and macular curvature, posterior segment length was univariably associated with macular curvature, but not after adjustment with age and sex, whereas spherical equivalent was negatively associated with the macular curvature coefficient after adjustment for age and sex (B = −6.19, 95% CI = −10.42 to −1.95, *P* = 0.004). In a sensitivity analysis, subfoveal choroidal thickness was negatively associated with macular curvature and might be another factor involved in macular shape development in children and adolescents aged 4 to 17 years.

These results regarding perinatal parameters extend previous knowledge that macular curvature is not only altered in adulthood due to ROP treatment^8^ but also in early life. This provides important data highlighting that either advanced ROP stages with need for treatment or treatment itself already lead to an altered macula curvature in early life. Although the genesis of how the flatter curvature in children with ROP-treatment develops is unknown, it can be speculated that laser treatment exerts tractional effects in the peripheral retina, resulting in a flatter or even negative macular curvature coefficient. Unfortunately, there were not enough participants treated with intravitreal injections to compare effects of laser versus intravitreal treatment on macular curvature, as the predominance of laser-treated eyes in our cohort reflects the standard of care during our study period (2003–2018), prior to the widespread adoption of anti-VEGF therapy for ROP.

The remaining negative effect of perinatal adverse events on the macular curvature coefficient in the multivariable models including ROP and ROP treatment suggests that additional remodeling processes in the eye may occur because of perinatal complications. Our participants with perinatal adverse events mostly had a history of bronchopulmonary dysplasia. The observed association with lower macular curvature may therefore reflect altered posterior segment development related to early-life oxygen exposure and systemic instability. Whereas choroidal alterations, including thinning^25^ have been described in this context, these do not fully explain the observed curvature changes. Additional mechanisms, such as disrupted retinal vascular development or altered scleral growth, potentially influenced by hypoxia and oxidative stress may also contribute.^26^ Due to highly unbalanced numbers of specific perinatal adverse events, these factors could not be analyzed separately in the present study.

In the cohort of adults born preterm, an association was observed between the macular curvature coefficient and posterior segment length.^8^ This association was also seen in the present cohort, but not after adjustment for sex and age. This may be explained by age-related axial elongation in this younger cohort, leading to a steepening of the macular curvature and an increase in the macular curvature coefficient. In adulthood, when growth has plateaued, interindividual variation in posterior segment length remained associated with curvature independent of age.^8^ This implies factors other than age-related development may contribute to an increase in the curvature coefficient in the elder cohort, perhaps factors such as myopic elongation and/or posterior staphyloma.^15^

We further observed that the subfoveal choroidal thickness was negatively associated with the curvature coefficient, even after adjusting for age and sex. This was similar in the adult cohort,^8^ and a negative correlation of the macular curvature coefficient and subfoveal choroidal thickness has also been found in adults with a mean age of 63 years.^15^ This implies a continuous relationship between macular curvature and subfoveal choroidal thickness across different age groups, suggesting that a decrease in choroidal thickness may contribute to an increase in macular curvature coefficient values, partially but not exclusively related to age. This might explain some of the myopic development in eyes with higher values of the curvature coefficient as seen in Supplementary Figure S2.

Further noticeable is the presence of negative curvature values, indicating a protrusion toward the vitreous cavity, as this also occurs in dome-shaped maculopathy.^6^ Legocki et al. reported an increased prevalence of dome-shaped maculae in preterm infants with ROP and pre-plus or plus-disease.^7^ In our cohort, however, the number of participants with negative curvature values was small. Although the descriptive distribution showed a higher proportion of negative values among children with treated ROP, the sample size was insufficient to further analyze this aspect. Moreover, most negative curvature coefficients were only slightly negative, indicating only mild protrusion.

The mechanisms underlying negative curvature coefficients may differ between ROP-treated non-ROP eyes. Peripheral retinal scarring from laser coagulation may contribute to a slight inward displacement of the posterior pole. In descriptive comparisons (Supplementary Table S1), subfoveal choroidal thickness was greater in eyes with negative curvature coefficients, suggesting subfoveal choroidal thickness might influence curvature in both sagittal directions. As choroidal thickness is influenced by multiple factors, including increased postnatal oxygen exposure,^25^ ocular perfusion^27^ and myopia control interventions,^28^ these factors may also have implications for macular curvature. However, our findings are descriptive and require confirmation in cohorts with more robust assessment of subfoveal choroidal thickness and a greater number of eyes with negative macular curvature coefficients.

The pathogenesis of DSM remains debated. Whereas increased subfoveal choroidal thickness has been associated with DSM in highly myopic eyes with posterior staphyloma,^29^ longitudinal studies suggest that the dome-shape results primarily from axial elongation in the perimacular region, rather than increased central submacular tissue.^30^^,^^31^

### Strengths and Limitations

The results from this study should be seen in context of its limitations. The study was conducted at a single study center and included primarily Caucasian participants, limiting generalizability. Not all eligible individuals could be contacted and although the overall cohort was large, subgroup sizes for ROP and ROP treatment were small. Findings for the group treated for ROP likely only reflect influences of laser-treatment and should not be generalized to other treatments such as anti-VEGF treatment. Further, curvature analysis was restricted to the immediate perifoveal region shown in a single horizontal foveal OCT scan. As wide-field imaging was not available, and the depth resolution was insufficient for scleral assessment, DSM or ridge-shaped macula could not be evaluated using a three-dimensional approach and parameters such as dome height were not assessed. Despite adjustment for ocular parameters including corneal power and axial length, residual OCT image distortions cannot be completely excluded.^12^ Analyses of specific perinatal adverse events, were also limited by small sample sizes.

Strengths of this study involve the large cohort of individuals born preterm across a broad age range and with varying degrees of prematurity, BW, ROP severity, and treatment. Extensive medical record review and a detailed questionnaire provided comprehensive information on perinatal conditions. Unlike in other studies^12^ that took a manual approach to extracting coordinates for a polynomial fit, the BM line here was automatically segmented and extracted. The advantages of using the BM line compared to the RPE line for curvature measurements have been explained in detail by Miyake et al.^32^ In addition, measurements were conducted by investigators masked to the participants’ birth status.

## Conclusions

This study found that among perinatal risk factors, treatment for ROP and perinatal adverse events were independently associated with macular curvature in childhood, resulting in smaller curvature coefficients and a flatter or slightly inward macular shape. This finding highlights that the effects of ROP treatment and perinatal adverse events on macular morphology already exist in early life.

A small number of eyes exhibited negative values of the curvature coefficient, but sample size limited further analysis. Associations between posterior segment length and the macular curvature coefficient appear to be mostly explained by age-related growth of axial length during ocular development. Descriptive analyses suggest a possible relationship between subfoveal choroidal thickness and curvature coefficients across age groups, indicating that choroidal thinning may contribute to macular shape changes.

## Supplementary Material

Supplement 1
