# Supplementary material for: Macular Curvature in Children Born Preterm With and Without ROP – Results From the Gutenberg Prematurity Study Young
Source: Invest Ophthalmol Vis Sci. 2026 Aug 11;67(10):27. doi: 10.1167/iovs.67.10.27 (PMC13480019; doi:10.1167/iovs.67.10.27)
Supplement: Supplementary file 1 [file iovs-67-10-27_s001.docx]

## Supplementary Material

#### Supplementary Table 1. Descriptive Distribution of Ocular Geometry, Visual Acuity and Spherical Equivalent in Eyes With Positive Versus Negative Macular Curvature

|  | **Positive macular curvature** | **Negative macular curvature** | **p** |
| --- | --- | --- | --- |
| n, Eyes (OD+OS) | 1,323 | 33 |  |
| DCVA (logMAR) (median [IQR]) | 0.00 [0.00, 0.10] | 0.00 [0.00, 0.20] | 0.312 |
| Spherical Equivalent (D) (mean (SD)) | 0.37 (1.46) | -0.06 (2.34) | 0.096 |
| Intraocular pressure (mmHg) (mean (SD)) | 16.49 (3.14) | 17.36 (2.45) | 0.142 |
| Central corneal thickness (µm) (mean (SD)) | 549.69 (34.89) | 555.10 (36.04) | 0.41 |
| Corneal radius (mm) (mean (SD)) | 7.80 (0.28) | 7.82 (0.40) | 0.825 |
| Anterior chamber depth (mm) (mean (SD)) | 3.19 (0.27) | 3.06 (0.37) | 0.021 |
| Lens thickness (mm) (mean (SD)) | 3.45 (0.18) | 3.49 (0.24) | 0.334 |
| Axial length (mm) (mean (SD)) | 23.08 (1.03) | 22.90 (1.50) | 0.331 |
| Posterior segment length (mm) (mean (SD)) | 16.49 (0.95) | 16.66 (1.31) | 0.404 |
| Foveal thickness (µm) (mean (SD)) | 281.02 (23.02) | 284.79 (29.37) | 0.387 |
| Subfoveal choroidal thickness (µm) (mean (SD)) | 318.26 (54.17) | 370.82 (74.09) | <0.001 |

OD – right eyes, OS – left eyes, n – number, DCVA – distant corrected visual acuity, D – diopters, mm – millimeters, mmHg – millimetre of mercury, SD – standard deviation, IQR – interquartile range

#### Supplementary Figure 1. Flowchart of Recruitment and Study Design

GA – gestational age, PT – preterm, ROP – retinopathy of prematurity, w – weeks

#### Supplementary Figure 2. Macular Curvature and Spherical Equivalent
